# Evolutionary pattern of pandemic influenza (H1N1) 2009 virus in the late phases of the 2009 pandemic.

**DOI:** 10.1371/currents.RRN1149

**Published:** 2010-03-03

**Authors:** Maria Beatrice Valli, Silvia Meschi, Marina Selleri, Paola Zaccaro, Giuseppe Ippolito, Maria Rosaria Capobianchi, Stefano Menzo

**Affiliations:** ^*^INMI "L. Spallanzani" Rome; ^†^INMI "L. Spallanzani" Rome, Italy and ^§^INMI "L. Spallanzani" Roma

## Abstract

Influenza A( H1N1)v has spread rapidly in all parts of the globe in 2009 as a true pandemic, although fortunately a clinically mild one. The relevant evolutionary steps for the new virus to adapt to human populations occurred very early during the pandemic, before the end of April. Of the several resulting clades or clusters, clade 7 appeared later and proved more successful, substituting all other early clades before the bulk of the worldwide infections occurred.

## **Introduction**

2009 influenza  A(H1N1)v pandemic virus has emerged following a recent reassortment event between swine strains [1] [2]. Its jump in the human population has been tentatively dated back to the beginning of the year [3] [4], and very early in its history the new virus could be differentiated in clades or, as later defined, clusters .[5] The significance of these findings is not clear, both in terms of a possible evolutionary pathway of the pandemic virus and in terms of pathogenicity. The early data showed that  clade 7 (as in ref. 4, or cluster 2 in ref. 5) appeared in New York a few weeks after clades 1 and 2 were isolated in Mexico and California, and originated late in March, but all clades were reported to co-circulate in all continents thereafter. After September, a second more intense peak has involved most temperate countries in  the Northern Hemisphere. However, viral sequence information on this second outbreak is relatively scant, and no clear trend in viral evolution has been outlined yet. In Italy most clades were circulating in the first months of the pandemic, when the great majority of the infections were imported by travellers (mostly from North and South America) who had become infected abroad. As in most European countries, a second, more intense wave of infections occurred in Italy during the period October-November 2009. Unlike the first epidemic peak of imported infections, this peak was powered by the rapid local spread of the virus (which had been circulating at low intensity during the whole summer) in children and adolescents (and their contacts) due to the opening of schools, kindergartens and other communities after the summer holidays.

## **Results**

We examined the nucleotide sequences (amplified from nasal swabs) of the hemagglutinin (HA, bases 440-828 of the coding sequence) and neuraminidase (NA) genes (variable length) from respectively 19  and 23 influenza A(H1N1)v strains isolated in the city of Rome, in the period May-August 2009. At position 658 (from the start codon) of HA the frequency of T was 63% (12/19), while the frequency of A (signature of clade 7/cluster2 virus) was 37%. These percentages were similar to those deduced from 589 HA sequences isolated globally (mostly in Mexico and in the United States) before August [5]. Viral strains isolated after September can be considered genuinely representative of the local evolution of the pandemic. The complete or partial HA and NA nucleotide sequences of an additional 43 influenza A(H1N1)v isolates were obtained. The frequency of the signature HA nucleotide 658 variants in those later isolates was 0% and 100% respectively for T and A, documenting the disappearance of other clades in favour of  clade 7. Among these sequences, no mutations considered to be biologically significant were detected, including the D222G/N (position 239 from the start codon in the H1N1 2009 pandemic virus) in HA and oseltamivir resistance mutations in NA, despite most patients had been subjected to treatment. Fig.1a and  b shows Neighbour-Joining phylogenetic trees (with bootstrap test) of HA and NA sequences (respectively) isolated at INMI from April to December 2009 (with indicated the month of collection) and compared to representative sequences of the initial pandemic from North America in April. 

### Figure 1a

**Figure:**
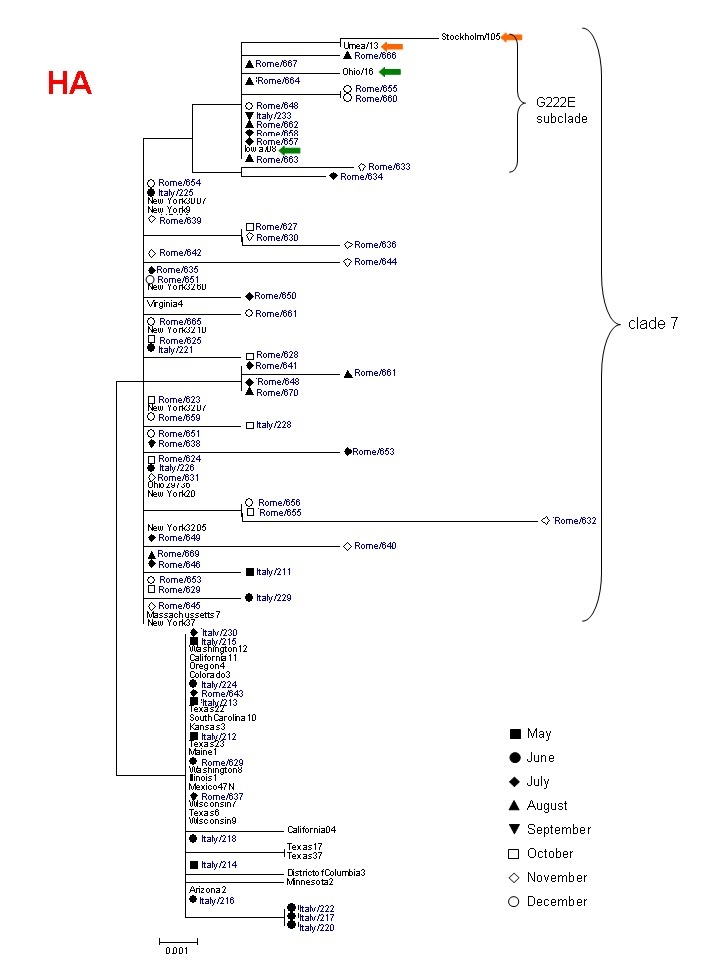
Phylogenetic tree of partial HA sequences from this study (in blue), indicating the month of collection, in comparison to sequences from North America. collected in April. Clade 7 and the HA G222E subclade are indicated. 4 additional HA G222E  sequences (collected after June) from USA  and Sweden, clustering with sequences from Rome, are indicated by green and orange arrows, respectively. The sequences from this study are indicated by the names deposited in GenBank, abbreviated  (Italy/xxx or Rome /xxx).

### Figure 1b

**Figure:**
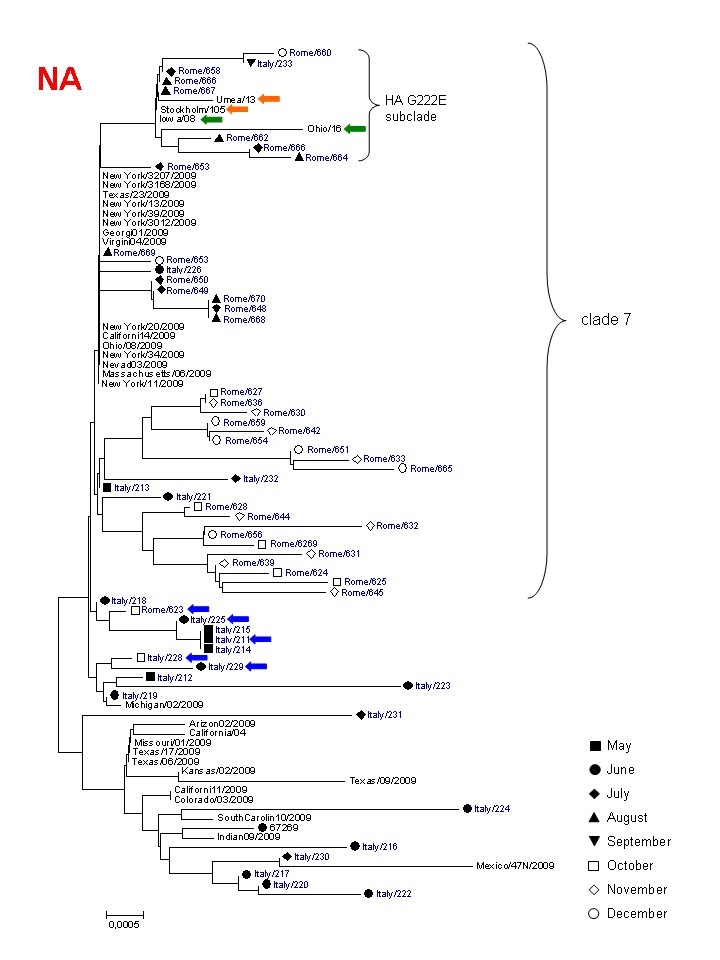
Phylogenetic tree of complete (with a few exceptions) NA sequences from this study (in blue), indicating the month of collection, in comparison to sequences from North America collected in April. Clade 7 and the HA G222E subclade are indicated. NA sequences from the same HAG222E isolates as in Fig 1a from USA and Sweden are indicated by green and orange arrows, respectively. Blue arrows indicate early clade 7  sequences from Rome clustering with non clade 7 sequences. The sequences from this study are indicated by the names deposited in GenBank, abbreviated  (Italy/xxx or Rome /xxx).

The HA tree confirms the clade shift during the summer. To be noted that a separate subclade of clade 7  consists of 12 sequences with the D222E substitution, which has been found at higher frequency in Italy, in Turkey and in Sweden, and whose biological meaning is still unknown. In contrast, a phylogenetic tree comparing NA sequences (full-length with a few exceptions), shows co-clustering of most clade 7  sequences with those from other clades until June, followed by progressive divergence of the late clade 7 Italian sequences from early New York sequences. By the end of June, clade 7 sequences from Rome isolates were clustering in three distinct subclades of clade 7. One of these was apparently the most successful as it  was associated with the  big autumn wave of infections. NA sequences from viruses bearing the HA G222E cluster together (including those from USA and Sweden), indicating that this mutation did not appear  by converging evolution of different strains but rather may represent a signature of an authentic subclade within clade 7 sequences.

To establish whether the significant clade shift was due to the local epidemic rather than a global phenomenon, and to identify the time course and the local trends of this evolution in different parts of the world, the total set of  HA sequences downloadable from GISAID database (December 2009) was divided by country (from countries with a reasonable number of sequences available over a period of at least three months) or geographical area and by month,  and analyzed at the signature nucleotide 658 in the HA sequence. There are a few limits to such a database analysis: 1) the geographical origin of the isolates does not necessarily indicate the actual origin of the infection, rather merely the origin of the infected person, especially for  early European and Asian isolates; 2) the collection times have been submitted with a variable degree of precision and some might be unreliable; 3) the majority of isolates have been sequenced in the first months of the epidemic, while very few sequences of later isolates have been published. Despite these limits, the pattern  of cluster substitution appears quite clearly everywhere. Fig. 2 shows the increasing proportion of clade 7 sequences in different countries from April to December. These patterns are completely superimposable if other signature nucleotides [4] [5] of clade 7/cluster2 virus are analyzed (not shown): NS position 367, MA positions 492 and 600, PB2 position 2163, confirming the stability of the clade and the absence of late reassortment events for these segments.

### Figure 2

**Figure:**
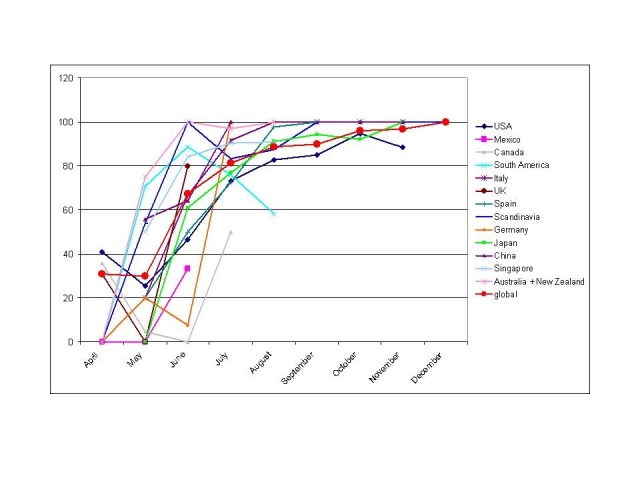
Proportion (%) of clade 7 sequences (HA) for each month in different countries or geographical areas.

The time frame of this phenomenon was different depending on the country. In the Southern hemisphere (Oceania and South America) clade 7 was already predominant by the end of April, reflecting perhaps the faster spread of the epidemic in the Southern (winter) part of the globe. In Singapore, China and Scandinavian countries the shift occurred a few weeks later, while in the majority of northern hemisphere countries it occurred mostly between June and July. The apparent fall of clade 7 sequences from April to May 2008 in the USA, UK and Canada, might be considered an artefact due to the overrepresentation of sequences from the New York clade 7 outbreak in April, which ignited the boom of scientific and public interest. The somewhat erratic behaviour of South American sequences (mostly from Brazil, Chile and Argentina) can be attributed to the fact that the aggregation of data from different countries  with such a huge North-South extension and with different timing in the epidemic peaks (necessary because of the low number of available sequences) might not be reliably representative of the whole area epidemic. The selection of clade 7 virus in Italian isolates (data from our lab, n= 66,  aggregated to those published by other labs,  n= 133 ) appears slightly anticipated compared to other Northern Hemisphere countries such as Mexico, USA, Japan, Spain. In USA and in Japan a few sequences  other than clade 7 were collected apparently as late as October/November.  

While the dynamics of the epidemics suggests a possible selective advantage of clade 7 virus over other early clades, the evolutionary trends within clade 7 need further investigation. One interesting analysis consists in the quantification of the selective pressure acting on the single genetic segments of the virus. For this purpose, 2 groups of clade 7 sequences (for each genomic segment) were selected, respectively collected in May (mostly from the initial New York outbreak) and in November (from all parts of the world, after the October peak). May sequences were randomly selected to match the much smaller number of  November sequences. The selective pressure acting on each segment  was computed as the ratio between the rate of non synonymous substitutions per non synonymous site and the rate of synonymous substitutions per synonymous sites (Ka/Ks) using the Nei and Gojobori substitution model ([6] with the Jukes-Cantor correction) implemented in the MEGA 4.0 package [7]. The Ka/Ks values suggest that a strong purifying selection was active on all segments, in agreement with previous findings for this and other influenza viruses [1] [8]. In particular, purifying selection was extreme (<0.1) on NP, MP, PA and PB1, moderate (>0.2) on NS and HA. To identify if this evolutionary pattern changed during the course of the pandemic, and to identify the period of the maximum positive selective pressure on the virus, the ratio of average pairwise Ka and Ks was computed separately within the May and the November groups of sequences (again for each segment), while the evolutionary step during the period May-November (that encompassed the greatest number of infections for most countries) was analyzed as the ratio of average pairwise Ka and Ks between each May and each November sequence (Fig. 3a). 

### Figure 3

**Figure:**
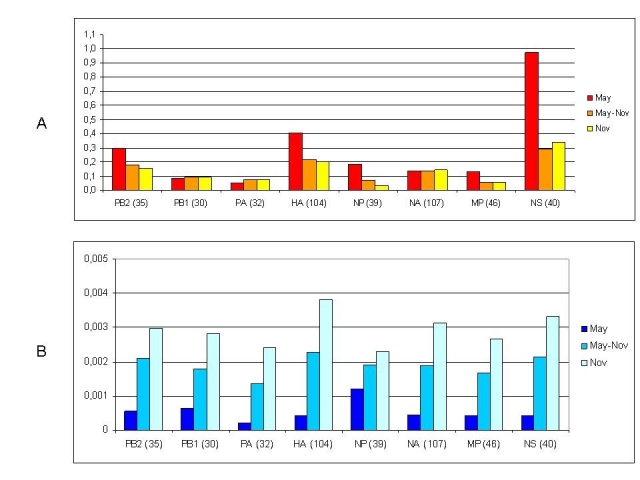
A) Average Ka/Ks values within May sequences, between May and November sequences and within November sequences, for each genomic segment. B) Nucleotide distances within and between the same set of sequences. For each segment, the number of sequences analyzed in the May group, which matches those in November, is indicated.

Two different patterns can be observed: for PB1, PA, and NA segments Ka/Ks remained very low and constant for the whole period; by contrast, for NS, HA , PB2, NP and  MP, Ka/Ks, although highly variable among segments, showed a common decreasing pattern. The dramatic reduction from May to November suggests that the strongest “positive” selective pressure acted before May on these segments. By contrast, the genetic distance values (Fig. 3b) for the same sets of sequences, indicate a progressive increase over time. Taken together, these findings suggest that the October-November peak of infections, which occurred in the Northern Hemisphere, did not impose any significant positive selective pressure on clade 7 virus, but only a random genetic drift in strict purifying selection conditions. 

## **Conclusions**

Our study demonstrates that pandemic (H1N1) 2009 virus has evolved worldwide, shifting from an initial mixed clade pattern to the predominance of one clade (clade 7) during the course of the pandemic. The virus constituting this clade was therefore responsible for most of the pandemic burden worldwide. After its origin, which remains obscure, clade 7 virus has been subjected to strong purifying selection, with the exception of the earliest phases of its evolution, behaving later as a well-fit virus, similar to viruses circulating in swine or seasonal influenza in humans. Interestingly, the highest Ka/Ks values were associated to HA and NS, key proteins for virus-host interactions, suggesting adaptation to the new host species. As yet, no pathogenetic correlate of this evolution has emerged, since no clear trend in the clinical aspects could be observed between the early and the late peaks of the epidemic [9]. Neither was a clear clinical impact demonstrated for HA variants which occurred on clade 7: D222/G/N or E, (WHO report, 28^th^ December 2009). The hypothesis that clade 7 virus enjoyed a marked advantage, in terms of transmissibility, over other early clades is intriguing, but has yet to be demonstrated.

## **Materials and Methods**

**Patients and samples**. Patients with febrile respiratory illness from the southern half of Rome were referred to the National Institute for Infectious Diseases (INMI) “L.Spallanzani” for diagnosis and treatment. All patients underwent nasal and faringeal swab sampling, and both swabs were stirred in the same tube containing RPMI 1640 tissue culture medium with antibiotics. Approximately half of the sequences in this study were obtained from patients with severe respiratory syndromes, the other half were from randomly chosen patients with mild symptoms. 

**RNA extraction, amplification and sequencing**. Nucleic acids were extracted from the swab fluid by an automated procedure (Biorobot MDx, Quiagen, Hilden, Germany) and amplified by in house methods using One-Step qRT-PCR system (Invitrogen, Carlsbad CA, USA) to yield partial or full-length sequences of HA and NA. Sequencing was performed on an automated ABI Prism 3130 instrument (Applied Biosystems, Foster City  CA, USA) by use of Big Dye3.1 cycle sequencing kits provided by the same manufacturer. All sequences have been deposited in Gen Bank with the following accession numbers: from CY052070 to CY052092 and from CY055309 to CY055414. 

**Phylogenetic and selective pressure analysis**. The sequences were aligned by the Clustal algorithm. Phylogenesis was performed using the MEGA 4 package [7].  The Neighbor-Joining phylogenetic trees (300 Bootstrap replicas) were generated using the Tamura 3 parameters distance option. The selective pressure acting on each segment was computed using the Nei and Gojobori substitution model ([6] with the Jukes-Cantor correction): Ka/Ks values were calculated as the ratio between the average rate of non synonymous substitutions per non synonymous site of all pairwise comparisons (average Ka), and the average rate of synonymous substitutions per synonymous site of all pairwise comparisons (average Ks). The genetic distances between sequences were calculated by the Tamura 3 parameter method.

## **Acknowledgments**

We gratefully acknowledge the editing of the manuscript by Carla Nisii.

## **Funding information**

This work has been partly supported by grants from the Italian Ministry of Health (Ricerca Corrente e Finalizzata).

## **Competing interests**

The authors have declared that no competing interests exist.

## References

[ref-846508738] Smith GJ et al. Origins and evolutionary genomics of the 2009 swine-origin H1N1 influenza A epidemic. Nature. 2009;459(7250):1122-5. 10.1038/nature08182 19516283

[ref-2412730914] Novel Swine-origin Influenza A (H1N1) Investigation team, Dawood FS et al. Emergence of a novel swine-origin influenza A (H1N1) virus in humans. N Engl J Med. 2009;360(25):2605-15. 10.1056/NEJMoa0903810 19423869

[ref-3649480668] Ranbaut A and Holmes E. The early molecular epidemiology of the swine-origin A/H1N1 human influenza pandemic. PLoS Curr Influenza. 2009 August 18:RRN1003 10.1371/currents.RRN1003 PMC2762654 20025195

[ref-218938320] Nelson M et al. The early diversification of influenza A/H1N1pdm. PLoS Curr Influenza. 2009 Nov 3:RRN1126 10.1371/currents.RRN1126 PMC2773564 20029664

[ref-381955057] Fereindouni SR, Beer M, Vahlenkamp T, Starick E. Differentiation of two distinct clusters among currently circulating influenza A(H1N1) viruses, March-September 2009. Euro Surveill. 2009;14(46) pii: 19409. 19941799

[ref-456803527] Nei M and Gojobori T. Simple methods for estimating the numbers of synonymous and nonsynonymous nucleotide substitutions. Mol Biol Evol. 1986;3(5):418-26. 10.1093/oxfordjournals.molbev.a040410 3444411

[ref-2140525700] Tamura K, Dudley J, Nei M and Kumar S MEGA4: Molecular Evolutionary Genetics Analysis (MEGA) software version 4.0. Mol Biol Evol. 2007; 24:1596-1599. 10.1093/molbev/msm092 17488738

[ref-282957631] Sinha NK, Roy A, Das B, Das S, Basak S. Evolutionary complexities of swine flu H1N1 gene sequences of 2009. Bioch. Bioph. Res Comm. 2009; 390: 349-351. 10.1016/j.bbrc.2009.09.060 19769939

[ref-2649623928] Donaldson LJ et al. Mortality from pandemic A/H1N1 influenza in England public health surveillance study. BMJ. 2009;339:b5213. 10.1136/bmj.b5213 PMC2791802 20007665

